# Functional IL-17R in glioma stem cells: potential role in tumor progression and therapeutic implications

**DOI:** 10.1186/2051-1426-3-S2-P412

**Published:** 2015-11-04

**Authors:** Prahlad Parajuli, Rohit Anand, Chandramouli Mandalaparty, Raviteja Suryadevara, Preethi Sriranga, Sharon Michelhaugh, Archana Thakur, Lawrence G Lum, Chaya Brodie, Sandeep Mittal

**Affiliations:** 1 Wayne State University & Karmanos Cancer Institute, Detroit, MI, USA; 2 Karmanos Cancer Institute, Wayne State University, Detroit, MI, USA; 3 Henry Ford Hospital, Detroit, MI, USA

## 

Interleukin 17 (IL-17) is one of the most potent inflammatory cytokines and has been strongly implicated in inflammatory autoimmune disorders like rheumatoid arthritis and multiple sclerosis. However, the role of IL-17 in tumor progression is quite controversial and the underlying mechanisms remain largely unknown. Our group and others have recently reported the prevalence of IL-17 secreting cells in malignant gliomas and studied the mechanism of their recruitment and immune functions in the tumor milieu. Immunohistochemistry and flow cytometry analysis of 14 tumor samples obtained from patients with malignant gliomas revealed that a considerable population (2-19% of total tumor cells) of all malignant gliomas expressed IL-17 receptor (IL-17R), with remarkable co-expression of the glioma stem cell (GSC) markers CD133, Nestin, and Sox-2. IL-17 enhanced the proliferation/self-renewal of CD133^+^IL-17R^+^ GSCs and also enhanced the number of GSC colonies in Matrigel® cultures. IL-17 also enhanced the expression of mesenchymal stem cell markers, as determined by qPCR analysis in GSC neurosphere cultures. Moreover, IL-17 induced cytokine/chemokine (IL-6, IL-8, interferon-γ-inducible protein [IP-10], and monocyte chemoattractant protein-1 [MCP-1]) secretion in GSCs, which were differentially blocked by antibodies to IL-17R and IL-6R. As determined by western blot analysis, treatment of GSCs with IL-17 enhanced the phosphorylation of signal transducer and activator of transcription 3 (STAT3), nuclear factor κ-light-chain-enhancer of activated B cells (NF-κB), and glycogen synthase kinase-3β (GSK-3β), while also enhancing β-catenin activity. IL-17R-mediated secretion of IL-6 and IL-8 were significantly blocked by inhibitors of STAT3 and NF-κB. On the other hand, NF-κB inhibitor was more potent than STAT3 inhibitor in blocking IL-17 induced MCP-1 secretion. Overall, our results suggest that IL-17–IL-17R interaction in GSCs potentially induces an autocrine/paracrine cytokine feedback loop, which in turn may provide an important signaling component for maintenance/self-renewal of GSCs via constitutive activation of both NF-κB and STAT3. This study provides novel insight into IL-17R-mediated inflammatory axis in glioma progression, which may have significant impact on clinical interventions in patients with malignant gliomas. Identification of IL-17R as a functional molecular target on the surface of GSCs, as presented in this study, could be a crucial first step for larger studies towards developing unique immune/therapeutic strategies aimed at either physical or functional attenuation of the GSCs.

